# Impacts of large-scale food fortification on the cost of nutrient-adequate diets: a modelling study in 89 countries

**DOI:** 10.1136/bmjph-2025-004613

**Published:** 2026-09-28

**Authors:** Leah Costlow, Yan Bai, Katherine P Adams, Ty Beal, Kathryn G Dewey, Christopher M Free, Valerie M Friesen, Mduduzi N N Mbuya, Stella Nordhagen, Florencia C Vasta, William A Masters

**Affiliations:** 1 Department of Agricultural, Food, and Resource Economics, Michigan State University, East Lansing, Michigan, USA; 2 Department of Food Science and Human Nutrition, Michigan State University, East Lansing, Michigan, USA; 3 School of Public Affairs, Zhejiang University, Hangzhou, Zhejiang, China; 4 Development Data Group, World Bank, Washington, DC, USA; 5 Institute for Global Nutrition, Department of Nutrition, University of California Davis, Davis, California, USA; 6 Global Alliance for Improved Nutrition, Washington, DC, USA; 7 Marine Science Institute, University of California Santa Barbara, Santa Barbara, California, USA; 8 Bren School of Environmental Science and Management, University of California Santa Barbara, Santa Barbara, California, USA; 9 Global Alliance for Improved Nutrition, London, UK; 10 Global Alliance for Improved Nutrition, Geneva, Switzerland; 11 Friedman School of Nutrition Science and Policy, Tufts University, Boston, Massachusetts, USA; 12 Department of Economics, Tufts University, Medford, Massachusetts, USA

**Keywords:** Public Health, Nutritive Value, economics

## Abstract

**Introduction:**

Large-scale food fortification (LSFF) is a widely accepted intervention to alleviate micronutrient deficiencies, yet policy implementation is often incomplete. Even assuming high implementation of existing mandatory and voluntary standards, the effects of LSFF on least-cost diets have not been established. Diet cost metrics such as the cost of nutrient adequacy offer insights into how policy interventions such as LSFF might change access to nutritious diets around the world.

**Methods:**

We estimated the extent to which LSFF reduces the retail cost of nutrient-adequate diets using retail food prices and fortification policy data from 89 countries. In total, we modelled 5874 complete pairs (without fortification vs with fortification) of least-cost diets across 22 sex-age groups and three nutrient-adequacy scenarios: meeting nutrient requirements only, adding minimum intakes for starchy staples and fruits and vegetables, and aligning food group shares with national consumption patterns. Single-vehicle and single-nutrient scenarios highlighted the cost reductions associated with specific fortification approaches. In addition, we used regression methods to estimate the relationship between diet cost reductions and the total quantity of nutrients added to the food supply.

**Results:**

Assuming 90% implementation of existing LSFF standards for wheat flour, maize flour, rice and oil, we found median retail cost reductions of 1.6%, 2.4% and 4.5% across the three scenarios. Cost reductions varied widely by sex-age groups, national fortification strategies and food price structures. Results from the regression analysis revealed that intermediate levels of food fortification resulted in the largest potential diet cost reductions.

**Conclusions:**

These findings highlight that LSFF may improve economic access to nutritious diets when policies are carefully designed for local contexts, making it a valuable complement to other efforts that improve access to nutritious diets.

**WHAT IS ALREADY KNOWN ON THIS TOPIC**

- Large-scale food fortification (LSFF) is a proven tool for reducing the prevalence of micronutrient deficiency at relatively low cost, but food supplies are often not in full alignment with existing mandatory and voluntary fortification standards.
- Nutrient-adequate and healthy diets are too expensive for many low-income people around the world to purchase, which suggests that LSFF could help improve nutritional status without imposing new cost burdens.

**WHAT THIS STUDY ADDS**

- In the first study of its kind, we estimated the reduction in the modelled costs of nutrient-adequate diets that would result from 90% implementation of existing LSFF regulations, with a baseline comparison scenario assuming no fortification and models capturing the varying nutritional needs of different population subgroups.
- Fortification could reduce the cost of nutritious diets, but the size of the reduction depends on how fortification policies are specified and on how expensive nutritious diets were prior to fortification, which is linked to underlying food price dynamics.

**HOW THIS STUDY MIGHT AFFECT RESEARCH, PRACTICE OR POLICY**

- Fortification policies should be viewed as occupying a complementary role alongside broader food system or market-based efforts to expand economic access to healthy diets.

## Introduction

 Micronutrient deficiencies affect billions of people worldwide.^1^ They compromise immune and endocrine function, impair cognitive development and increase the risk of maternal and child morbidity and mortality.^2^ Despite advances in global health and nutrition, such deficiencies persist, particularly among vulnerable populations including young children and women of reproductive age.^3^ Addressing micronutrient gaps at the population level is thus a global health imperative. Large-scale food fortification (LSFF), which involves adding essential vitamins and minerals to widely consumed staple foods, beverages, or condiments, has emerged as an effective strategy for reducing micronutrient deficiencies at the population level.^4–8^ Yet while the health benefits of LSFF are well documented, its potential to lower the cost of nutrient-adequate diets and improve economic access to adequate nutrition has received limited attention.

Policies to standardise the fortification of commonly consumed staple foods or condiments have been widely instituted in countries across all income levels to ensure sufficient intake of essential nutrients that might otherwise be under-consumed in average diets.^4,9,10^ However, biomarker data and dietary intake data show that micronutrient deficiencies remain prevalent,^1,3^ and in many countries, existing LSFF mandates have not been fully implemented, while in other cases fortification standards are voluntary and industry participation may be low.^11^ When food supplies do not comply with fortification standards, nutrient sufficiency can only be achieved by consuming larger quantities of nutrient-rich foods, including fruits, vegetables, and animal-source foods, which tend to be more expensive per unit of energy than fortified or unfortified staple foods.^12^ Healthy diets that are aligned with food-based dietary guidelines provide essential nutrients in the recommended amounts, but remain unaffordable for nearly 3 billion people around the world^13–15^ and are more expensive than the cost of diets that meet nutrient requirements without necessarily providing sufficient quantities of all recommended food groups. This context highlights the importance of assessing whether LSFF can serve as a cost-effective strategy to increase the affordability of nutrient-adequate diets. Fortification could make low-cost staple foods more expensive if implementation costs are high and if high pass-through rates result in higher retail prices for consumers. Conversely, fortification could also succeed in providing low-cost sources of otherwise costly nutrients, resulting in least-cost diets that meet nutrient requirements at a lower overall cost.

Past research has established the health benefits of LSFF,^7^ and numerous modelling studies have investigated the cost-effectiveness or benefit-cost ratios of LSFF implementation in countries around the world.^16^ However, there has not yet been a comprehensive study combining globally comparable price and fortification policy data and using linear optimisation models to estimate the role of LSFF in improving economic access to nutritious diets. Our analysis addresses this gap by building on least-cost diet modelling methods that have previously been used to identify cases where key nutrients are unusually expensive to include in the diet, to identify differences in the cost of meeting nutrient needs across the life cycle and to highlight seasonal variations in nutrient costs.^17–19^ The base scenario in this analysis is the cost of a nutrient-adequate diet, whose value for a reference adult female has been monitored by the World Bank as a global indicator, with estimates now reported across countries to inform food and nutrition security monitoring efforts.^20^ This study adapts these modelling tools to estimate the extent to which more complete implementation and enforcement of country-level LSFF policies would reduce the cost of nutrient-adequate diets and improve food and nutrition security around the world.

In this analysis, we calculated the cost of nutrient adequacy (CoNA) first assuming zero LSFF implementation and second assuming 90% implementation to estimate the reduction in diet costs due to fortification across three variations on a nutrient-adequate diet. The term ‘implementation’ is used to describe compliance, that is, the extent to which food processors comply with mandatory and voluntary fortification policies and produce foods that are fortified to specified levels. This provides insights into how implementation of fortification standards might improve access to nutrient-adequate diets and whether existing standards are optimally designed for this purpose. We estimated a total of 5874 diet cost reductions for 89 countries and 22 population subgroups, iterating across three model specifications: (1) a basic model that identifies least-cost diets meeting nutrient requirements by subgroup, (2) an extension that additionally requires minimum intake of starchy staples (SS) and fruits and vegetables (FV) and (3) a further extension that sets minimum energy shares from major food groups reflecting consumption patterns in each country based on Supply Utilization Accounts (SUA) from FAO’s Food Balance Sheets.^21^ All models used the most recent 2021 global retail food prices from the International Comparison Program,^22^ coordinated by the World Bank, and LSFF policy data through 2022 from the Global Fortification Data Exchange (GFDx).^10^

## Materials and methods

### Diet cost models

We calculated CoNA across three modelling scenarios using linear programming in a method adapted from previous publications.^17,19^ This method minimises the total cost of a food basket while ensuring compliance with a set of nutritional constraints, including targets for macronutrients, micronutrients and total dietary energy. We updated the original CoNA method to include food group constraints in two additional scenarios. In all three scenarios, we included nutrient constraints for intakes of macronutrients and essential micronutrients (see online supplemental appendix table A1 for a complete list). Nutrient requirements for 22 subgroups by age, sex, pregnancy status and lactation status (hereafter referred to as ‘population subgroups’) were from the Harmonized Nutrient Reference Values as defined in Allen *et al*^23^ and included average requirement and Tolerable Upper Intake Levels (ULs) for each nutrient as relevant. Diets meeting the needs of children under the age of 4 years were not included in this analysis. Energy requirements were calculated to align estimates from the European Food Safety Authority^24^ with as defined in Allen *et al*^23^ and assumed moderate physical activity (PAL=1.8).

All three models included the same core constraints of nutrient adequacy. In the first scenario (CoNA), modelled diet costs were calculated to meet basic nutrient requirements with no additional food group requirements. This scenario represents the lower bound of nutrient adequacy by meeting nutrient needs without respect for food-based recommendations or cultural preferences. However, since basic nutrient adequacy does not result in modelled diets that broadly conform to culinary or cultural expectations about a balanced diet, including items from a range of particular food groups, we assessed two additional scenarios incorporating food group constraints to ensure that a given amount of energy from specified food groups was included in each modelled diet.

In the second scenario (CoNA-SS&FV), modelled diets were additionally required to meet a minimum threshold for energy from fruits and vegetables (combined, abbreviated as FV) and starchy staples (SS). Minimum intakes for these food groups were derived from the Healthy Diet Basket (HDB), which reflects commonalities across international food-based dietary guidelines and serves as the basis for global monitoring of the cost of a healthy diet (CoHD).^14^ The HDB provides reference intakes in kilocalories per day for a healthy, non-pregnant, non-lactating 30-year-old woman. We scaled target food group intakes up or down to accommodate changes in dietary energy requirements for other population subgroups. This scenario represents the cost to purchase nutrient-adequate diets while also satisfying the core nutrition-sensitive requirements of food-based dietary guidelines.

In the third scenario (CoNA-SUA), modelled diets instead were required to provide energy from four food groups recommended in the HDB, but within lower and upper bounds of national average consumption rather than in the quantities recommended for health. This scenario included food group constraints for fruits and vegetables (combined); starchy staples; legumes, nuts, and seeds (combined); and animal-source foods. Food supply quantities of energy from each food group were calculated at the country level from the FAO SUA^21^ and converted to energy shares using the daily energy requirements of each population subgroup. Upper and lower bounds for food group energy shares are calculated as the interquartile range (25th through 75th percentiles) of the food supply reported between 2010 and 2022. This scenario represents the cost to purchase nutrient-adequate diets that broadly conform to existing country-level consumption patterns by food group, assuming that food group energy shares are consistent across population subgroups.

For each of the three modelled diet scenarios, we first estimated diet costs assuming no fortification; then recalculated diet costs under the LSFF implementation scenario with increased nutrient density for fortified foods; then calculated the change in diet costs attributable to fortification. In each case, resulting modelled diet costs reflect standard matching of food composition data to non-fortified items. Foods that are potentially subject to fortification (‘food vehicles’) include rice, oil, maize flour, and wheat flour, and were identified as fortifiable or not fortifiable at the country level based on the documentation of mandatory or voluntary fortification standards in GFDx. Retail food items were manually matched to food vehicles as appropriate based on item descriptions (see online supplemental appendix table A2 for examples of the matching process).

Our modelling of existing fortification policies depended on several key assumptions related to (1) fortification policies and (2) premix costs. Although ready-made foods may include fortified ingredients, this varies widely by country and is not documented in GFDx. Accordingly, we assumed that packaged, processed and other ready-made foods with the exception of white bread are not subject to fortification, even if they may contain ingredients that are matchable to fortifiable food vehicles in a given country. For example, bagged wheat flour, white bread, and bottled vegetable oil were considered potentially fortifiable while whole grain bread, crackers, and sardines packed in oil were not. In most countries whole wheat flour is not subject to fortification based on GFDx documentation, with the exception of atta in India and Bangladesh. Fortified salt was excluded from this analysis and thus not allowed to enter into any of the modelled diet scenarios, along with all other non-caloric food items, spices, condiments, beverages, and foods that are not recommended as part of a healthy diet, such as sweets, snacks, and cured meats.

### Regression model

We used a regression approach to describe how diet cost reductions are associated with baseline diet costs and with intensity of fortification policy, where policy intensity is estimated as the sum of all potentially fortifiable nutrients across all food vehicles in each country. This section of the analysis supplements the main diet cost modelling results and provides insights into whether countries that add larger quantities of nutrients to the food supply have larger potential cost reductions. A simple quadratic ordinary least squares (OLS) regression model reflects the likelihood that higher levels of fortification provide greater cost reductions up to an inflection point, after which the addition of further nutrients to the food supply is no longer efficient and results in higher costs due to the sensitivity of ULs for certain nutrients. All models included fixed effects for diet cost model and geographic region as shown in Equation 1:


(1) $$∆C=\beta_{0}+\beta_{1}X_{i}+r_{i}+m_{i}+e_{i}$$

where ∆C is the estimated percentage change in diet costs, $X_{i}$ is either the intensity of fortification or baseline diet costs in country *i*, and $r_{i}$ and $m_{i}$ are country-level fixed effects for World Bank region and diet cost model. Policy intensity was represented as the total combined amount of all fortified nutrients added across all food vehicles in each country, measured in mg per kg.

### Data

We used retail food prices from 2021 from the International Comparisons Program (ICP), coordinated by the World Bank.^22^ This dataset includes 173 countries and was made available to the Food Prices for Nutrition project under a confidential agreement with the World Bank. Retail prices from ICP represent globally and regionally standardised item lists for cross-country comparisons. Information on mandatory and voluntary fortification policies is available for 154 countries from the GFDx,^10^ including data on the food vehicle, nutrient and fortification amounts specified in LSFF policies in each country. National food supply data are available from FAO for 190 countries in total between 2010 and 2022. The final analytical sample of countries with data from all three data sources included 89 countries (see online supplemental appendix table A4 for a complete list of countries in the analysis). Estimated reductions in diet costs are presented as global medians due to measurement error in country-level retail food prices and to account for notable outliers in the estimated reductions. Country inclusion and data on fortification coverage by food vehicle and micronutrient are summarised in the online supplemental appendix table A5.

Implementation of existing LSFF policies varies widely across countries and within countries across food vehicles.^10^ We modelled a hypothetical fortification scenario in which implementation of fortification mandates achieves 90% compliance with all documented policies, consistent with the improved compliance fortification scenarios modelled in Friesen *et al*.^7^ Current implementation of fortification standards falls short of this 90% threshold on average, even for mandatory standards; while implementation approaches 100% for some mandatory standards in some places, typical levels of implementation range between 25% and 80%.^7^ To calculate diet costs with LSFF implementation, we adjusted food composition for foods subject to fortification standards by adding 90% of the fortification amount to the baseline content of the fortified nutrient. In cases where multiple fortification standards are documented in GFDx for the same nutrient in a single country, we used the lowest reported level as a single decision rule, resulting in a conservative estimate of the lower-bound for potential fortification. In countries where wheat flour is fortified, we assumed that fortified wheat flour accounts for 75% of fortified white bread by weight, an estimate that is aligned with previous LSFF modelling studies.^25^ The difference in cost between LSFF implementation and baseline scenarios represents the cost reduction attributable to LSFF. All costs were converted to 2021 international dollars using purchasing power parity conversion factors.^22^ We accounted for implementation costs by adding the cost of producing fortification nutrient premix to the ICP retail price for foods subject to fortification, assuming 100% pass-through of premix costs to retail prices which results in a higher-bound estimate of the effect of fortification on food prices. However, premix costs are low overall and account for a mean value of 0.1% of retail food prices across all food vehicles (see online supplemental appendix figure A4). Premix costs were estimated at the country and food vehicle levels and account for micronutrient type, fortification amount, chemical compound (fortificant), and supply chain costs as reported in Friesen *et al*.^7^ We did not estimate the additional implementation and administrative costs that the food industry or national governments would likely incur. See online supplemental appendix table A6 for results of a sensitivity analysis using alternative pass-through (50%) and LSFF implementation (50%) rates.

## Results

Implementation of existing country-level fortification policies resulted in reduced diet costs on average across all subgroups included in the analysis (see online supplemental appendix table A3 for average regional cost results). Diet cost estimates were successfully obtained for nearly all cases, but solutions were infeasible in 0.6% of all unique country-subgroup-scenario-fortification combinations (37 out of 11 748; see online supplemental appendix table A7 for a list of cases with no solution). Without fortification, CoNA had the lowest median daily cost at $2.70 PPP (IQR: $2.20 to $3.23) across all countries and population subgroups. The addition of two food group constraints in the CoNA-SS&FV scenario raised the median daily cost to $3.09 PPP (IQR: $2.52 to $3.83). The CoNA-SUA scenario had the highest median daily cost at $3.60 PPP (IQR: $2.97 to $4.52). In comparison, the CoHD meeting food-based dietary guidelines was a median of $3.48 PPP (IQR: $3.05 to $3.88) according to official 2021 estimates.^20^ The relatively higher cost of the CoNA-SUA scenario reflects wide variation in observed food group consumption shares, as well as potential selection effects due to the smaller sample size of the present analysis.

Implementation of LSFF at 90% of existing standards reduced diet costs for CoNA by an average across all countries and population subgroups of $0.04 PPP or about 1.6% per day (IQR: −2.4% to 0%), for CoNA-SS&FV by $0.08 PPP or about 2.4% per day (IQR: −4.3% to 0%) and for CoNA-SUA by $0.16 PPP or about 4.5% per day (IQR −8.8% to −0.1%). In figure 1, panel A depicts the range of changes in the cost of a nutrient-adequate diet across all 89 countries in the analytical sample, while panel B depicts the range of cost changes among only the subset of countries with a non-zero change in diet costs under LSFF implementation (see within-figure labels for sample size by scenario). Within the 5874 modelled diet pairs across 89 countries, 22 population subgroups, and three scenarios, fortification reduced diet costs in 55.1% of modelled diets, had no effect in 38% and led to higher diet costs in 7%, while no feasible solution was obtained in 0.6% (37 cases). Within the full analytical sample, 90% implementation of fortification standards was responsible for a very small median cost reduction across all population subgroups and modelled diet scenarios of 0.1% (IQR: −4.6% to 0%). Among only those countries and scenarios with non-zero cost changes, median reductions were larger at 2.4% (IQR: −9.0% to −0.3%).

**Figure 1:**
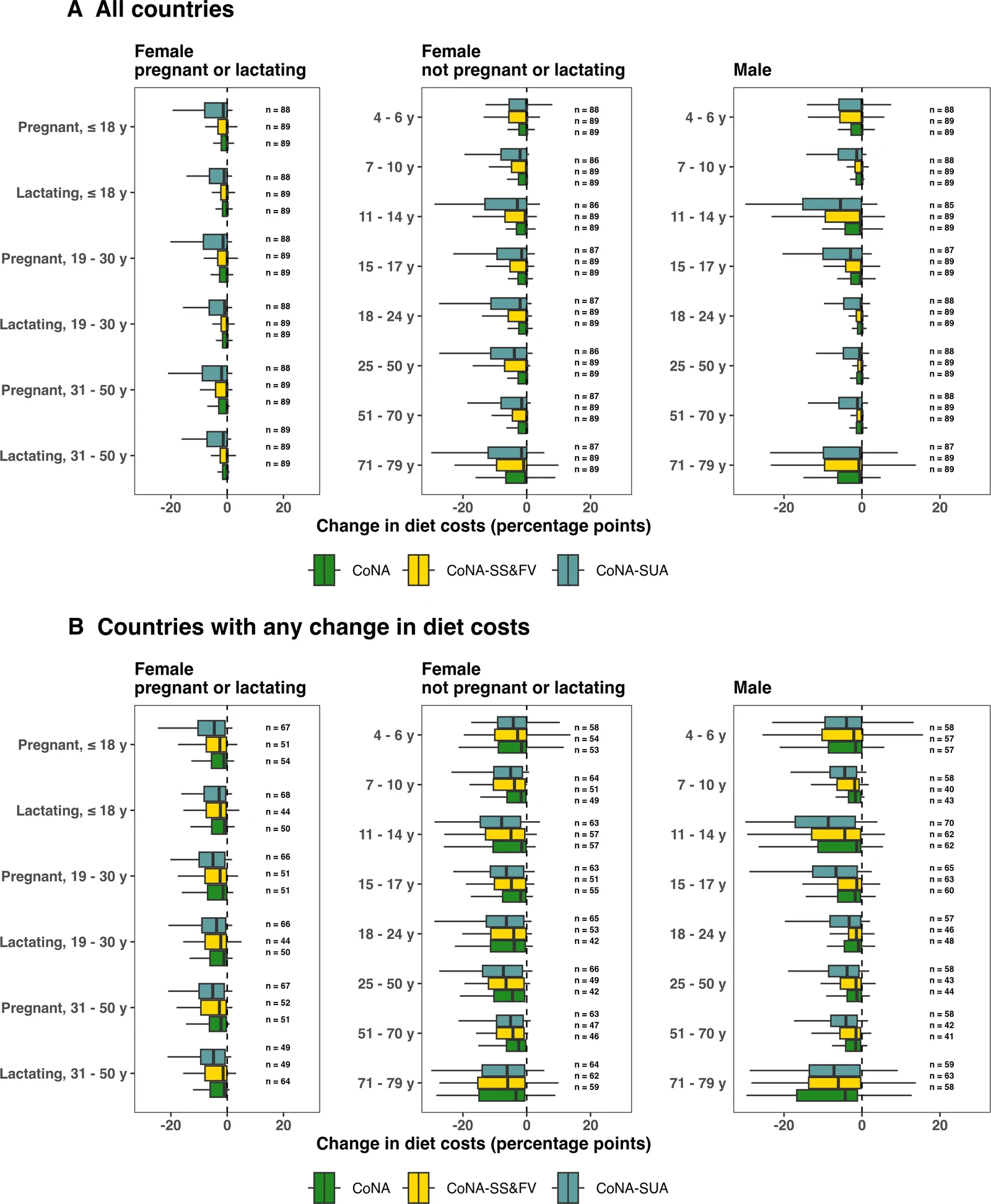
Change in cost of nutrient-adequate diets with LSFF implementation by scenario and population subgroups. Data shown in panel A are changes in diet costs from all countries in the analytical dataset with non-missing solutions to all modelled diets, while data shown in panel B are changes in diet costs from a varying subset of countries with non-zero change in diet costs under LSFF implementation. Negative values indicate reduced costs with LSFF while positive values indicate increased costs with LSFF. CoNA refers to diets meeting nutrient requirements; CoNA-SS&FV refers to diets that also meet basic healthy food group constraints for starchy staples and for fruits and vegetables (combined); and CoNA-SUA refers to diets that align with national average consumption patterns at the food group level. Outliers are omitted from all boxplots using the Tukey standard (1.5 × IQR), as are 37 cases with no feasible solution. CoNA, cost of nutrient adequacy; FV, fruits and vegetables; LSFF, large-scale food fortification; SS, starchy staples; SUA, Supply Utilization Accounts.

Implementation of LSFF resulted in larger diet cost reductions for certain population subgroups compared with others (figure 1). For countries where implementing LSFF results in a change in diet costs, median reductions were slightly larger for female subgroups at 2.6% (IQR: −9.5% to −0.3%) compared with 2.1% (IQR: −8.1% to −0.2%) for male subgroups. Within female subgroups, median cost reductions were smallest during lactation at 1.7% (IQR: −7.3% to −0.2%), larger during pregnancy at 2.2% (IQR: −8.4% to −0.3%), and largest for females of all ages who are not pregnant or lactating at 3.2% (IQR: −10.7% to −0.3%). The range of reductions was widest for females of all ages, boys aged 4–6 years, adolescent boys aged 11–14 years and men aged 71–79 years.

Cost reductions also varied across the three model specification scenarios, although there was substantial overlap. Under the nutrients-only CoNA model, median cost reductions ranged from 0.5% for girls 18 years old and younger during lactation and pregnancy to 3.4% for men aged 71–79 years. Under the CoNA-SS&FV model, median reductions ranged from 0.7% for boys aged 15–17 years to 5.1% for women aged 25–50 years. The largest reductions occurred under the CoNA-SUA model, with median reductions ranging from 2.6% for girls 18 years old and younger during lactation to 7.3% for girls aged 11–14 years.

Median cost changes at the subgroup level were uniformly negative (ie, LSFF implementation consistently resulted in reduced rather than increased diet costs). Positive values located outside of the interquartile region of the boxplots (ie, on the whiskers) in figure 1 reflect country-subgroup combinations where fortification resulted in higher diet costs. This may result from two main causes in different cases: first, in some cases fortification of nutrients with ULs on their recommended consumption may lead modelled diets to include quantities of more expensive, non-fortified foods in order to meet minimum requirements for other nutrients while remaining below the UL for the fortified nutrient. Underlying patterns in country-level retail prices also contribute, as there may not be a substantial increase in diet costs if the alternative item is similarly low cost.

Across all three scenarios, reduced diet costs resulted from changes to the composition of the least-cost food basket (figure 2). Substitution between fortified and unfortified versions of the same items was the primary driver of reduced diet costs, with only minimal additional changes in the composition of modelled diet baskets. The increased nutrient density of fortified foods means that these items were included in large quantities, while the inclusion of unfortified staple foods decreased. In cases where non-fortified item inclusion increased with fortification, this indicates that the item did not contribute nutrients at minimum cost in the baseline CoNA scenario, and that the introduction of fortification provided greater flexibility in the linear programming algorithm for different foods to be included. For example, broad beans, spotted beans, mangoes, chicken, and milk are all included in fortified diet baskets to a larger degree than in unfortified diet baskets. The inclusion of other legumes, vegetables, and animal-source foods declined to a degree that largely offsets increases elsewhere such that, on average, food group energy remained roughly constant both with and without fortification (see online supplemental appendix figure A1). This is possible because the modelled diets include specified food groups in amounts that are at least equal to benchmarks, allowing for variation in food group energy across scenarios.

**Figure 2:**
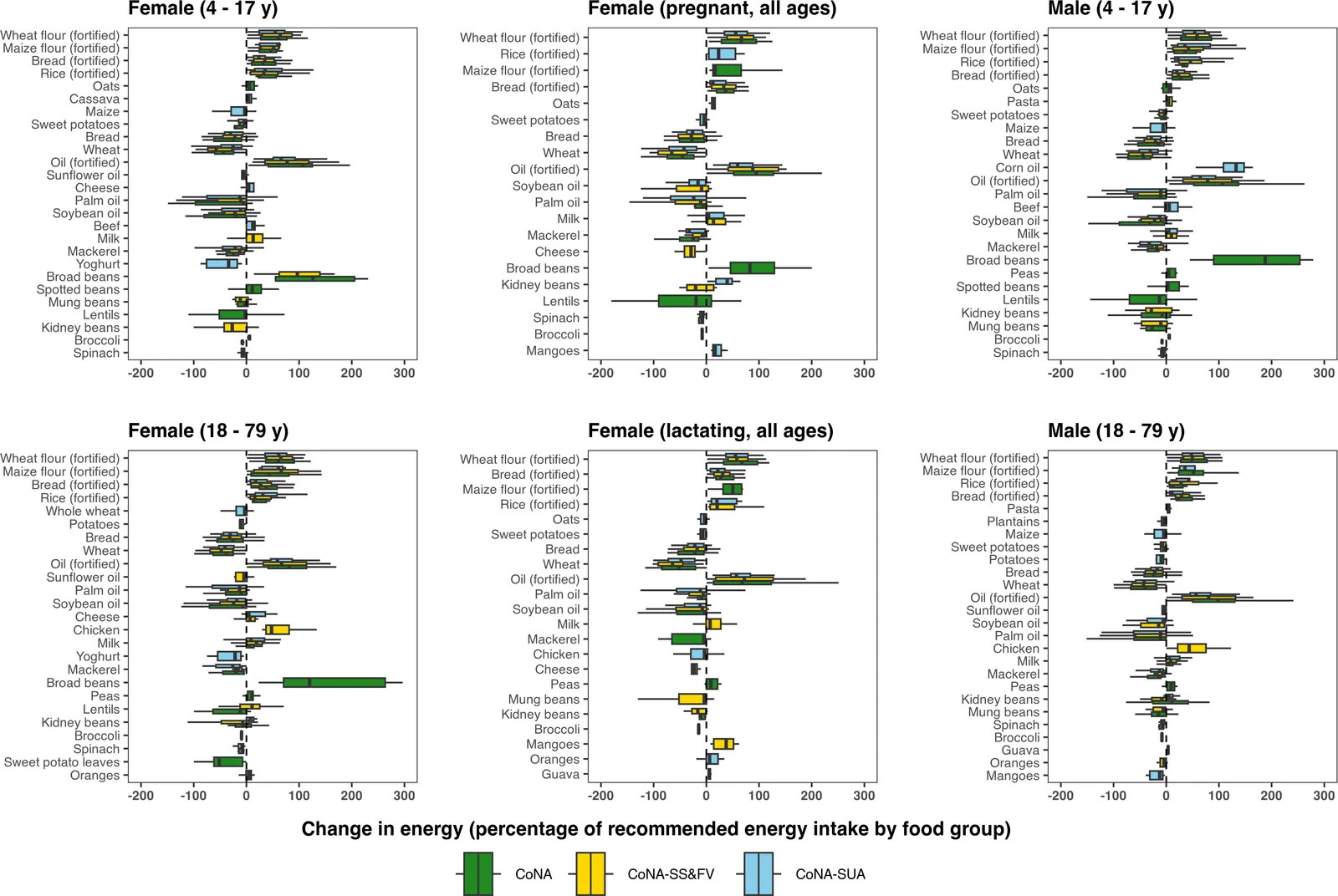
Changes in modelled diet composition by item with LSFF implementation by scenario and population subgroup (percentage of recommended energy intake by food group). Data shown are changes in average energy from items included in least-cost diets as a percentage of recommended energy intake by food group for each population subgroup, while population subgroups are combined for ease of presentation. Negative values indicate reduced costs with LSFF while positive values indicate increased costs with LSFF. Recommended intakes are derived from the Healthy Diet Basket and are scaled up or down for each subgroup’s energy needs. Items whose median energy included changes by 3% or less under fortification are excluded from the visualisation. CoNA refers to diets meeting nutrient requirements; CoNA-SS&FV refers to diets that also meet basic healthy food group constraints for starchy staples and for fruits and vegetables (combined); and CoNA-SUA refers to diets that align with national average consumption patterns at the food group level. Outliers are omitted from all boxplots using the Tukey standard (1.5 × IQR). CoNA, cost of nutrient adequacy; FV, fruits and vegetables; LSFF, large-scale food fortification; SS, starchy staples; SUA, Supply Utilization Accounts.

Diet cost reductions were primarily driven by certain food vehicles more than others (figure 3). These results reflect the isolated effect on diet costs of fortifying only one food vehicle at a time. Fortification of wheat flour, which was the most commonly fortified food vehicle (see online supplemental appendix table A5), contributed the largest reduction in diet costs across all three scenarios and for all population subgroups. Rice fortification contributed to a lesser extent but still consistently led to diet cost reductions for most subgroups, especially in the CoNA-SUA scenario. On average, oil fortification had no effect on diet costs across all scenarios and subgroups, while maize flour fortification had no effect in most cases and led to higher diet costs for boys aged 4–6 years in the CoNA-SS&FV scenarios.

**Figure 3:**
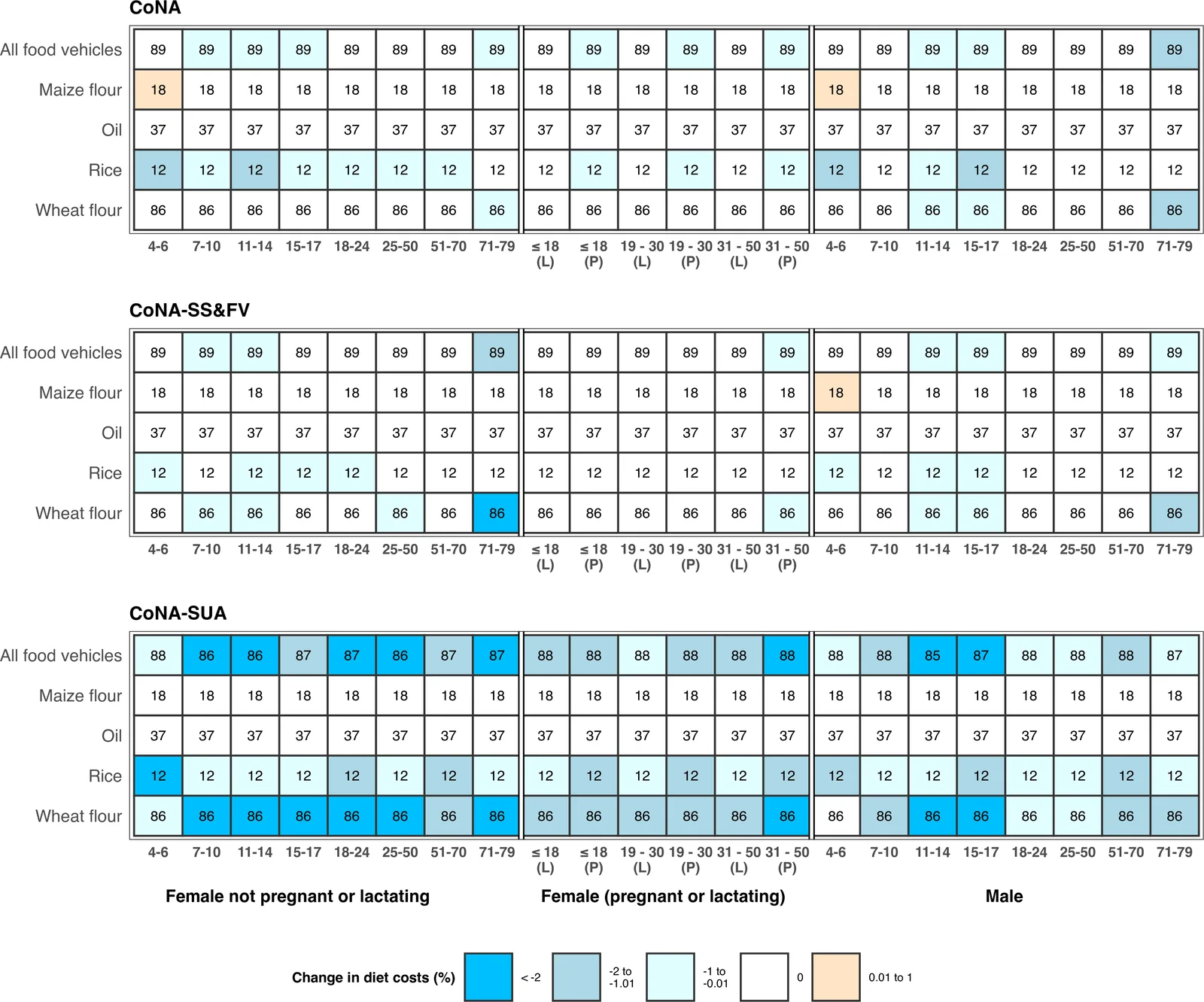
Median percentage change in cost of nutrient-adequate diets with LSFF implementation by food vehicle and population subgroup. Data shown are median reductions in diet cost by scenario, food vehicle and population subgroup. Labels indicate sample size (n=countries). Numbers on the y-axis indicate age ranges in years. Cost reductions are calculated as percentage change in 2021 international dollars (PPP). CoNA refers to diets meeting nutrient requirements; CoNA-SS&FV refers to diets that also meet basic healthy food group constraints for starchy staples and for fruits and vegetables (combined); and CoNA-SUA refers to diets that align with national average consumption patterns at the food group level. Cases with no feasible solution are omitted. CoNA, cost of nutrient adequacy; FV, fruits and vegetables; LSFF, large-scale food fortification; PPP, purchasing power parity; SS, starchy staples; SUA, Supply Utilization Accounts.

Changes in diet costs under LSFF implementation were also driven by certain nutrients more than others, while other nutrients contributed to increased diet costs (figure 4). As in figure 3, these results reflected the isolated effect on diet costs of fortifying one nutrient at a time. Fortification with calcium drove cost reductions in the CoNA and CoNA-SS&FV scenarios, with some contribution from vitamin E. Fortification with iron contributed to the slightly larger diet cost reductions in the CoNA-SUA scenario, again with some contribution from vitamin E. Fortification with selenium led to diet cost increases across all three scenarios, although only four countries had selenium fortification policies. Zinc fortification caused sporadic diet cost increases as well. For the remaining nutrients, median reductions were zero, indicating that single-nutrient fortification had no effect on least-cost diets.

**Figure 4:**
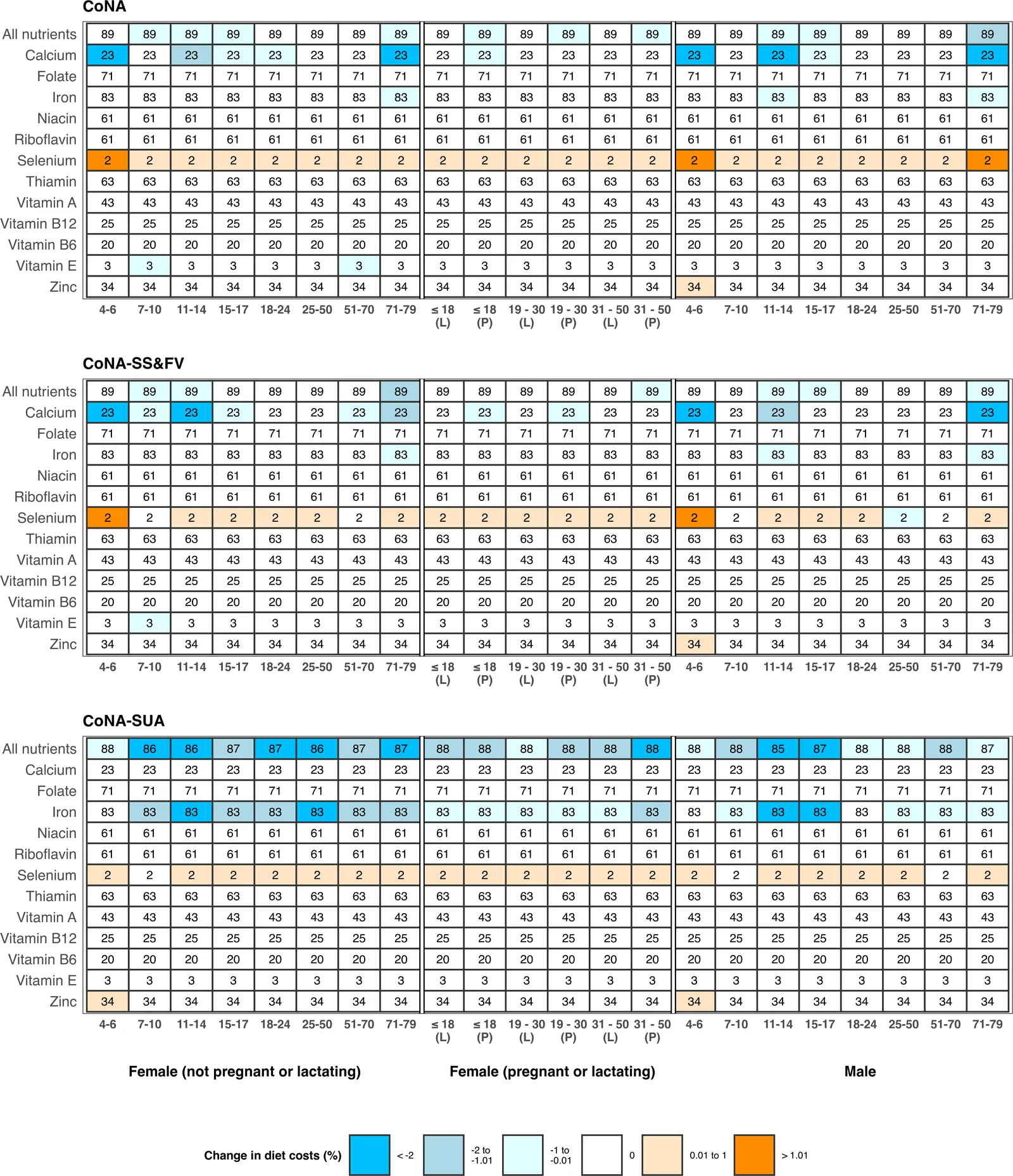
Median percentage change in cost of nutrient-adequate diets with LSFF implementation by nutrient and population subgroup. Data shown are median reductions in diet cost by scenario, nutrient and sex-age subgroup. Labels indicate sample size (n=number of countries). Numbers on the y-axis indicate age ranges in years. Cost reductions are calculated as percentage change in 2021 international dollars (PPP). CoNA refers to diets meeting nutrient requirements only; CoNA-SS&FV refers to diets that also meet healthy food group minimum intakes for starchy staples, fruits and vegetables; and CoNA-SUA refers to diets that align with national average consumption patterns at the food group level. Cases with no feasible solution are omitted. CoNA, cost of nutrient adequacy; FV, fruits and vegetables; LSFF, large-scale food fortification; PPP, purchasing power parity; SS, starchy staples; SUA, Supply Utilization Accounts.

Diet cost reductions across all three model scenarios had a non-linear relationship with the intensity of a country’s fortification policies and a largely negative association with baseline diet costs, as shown in figure 5. Policy intensity was estimated as the sum of all potentially fortifiable nutrients across all food vehicles in each country. Countries with the largest reductions in diet costs tended to have intermediate combined fortification amounts (left-hand column), whereas those with the most extensive combined fortification amounts were more likely to have no cost reductions or even diet cost increases. Similarly, reductions were often greatest in countries with relatively high baseline diet costs prior to LSFF implementation (right-hand column). However, these correlations were not strictly consistent, as a wide range of diet cost reductions was observed across the distribution. In addition, diet cost reductions were further linked with geographic region. Countries in Latin America and Caribbean tended to have both higher diet cost reductions, intermediate fortification amounts and relatively higher baseline diet costs. Countries in sub-Saharan Africa tended to have lower or no diet cost reduction, higher fortification amounts and more moderate baseline diet costs. Fortification resulted in no change in diet costs for most countries in East Asia and Pacific or in Europe and Central Asia, although fewer European countries were included in the analytical dataset. Across all population subgroups, average cost reductions were largest for high-income and upper middle-income countries (online supplemental appendix figure A5). However, due to the small sample size of the analysis and selection effects resulting in a low representation of high-income countries, these region-level and income-level associations should be viewed cautiously. Underlying food price patterns may also be responsible for patterns in diet cost reductions; for example, if nutritious foods are already inexpensive in a given country, then LSFF implementation might not result in meaningfully lower modelled diet costs.

**Figure 5:**
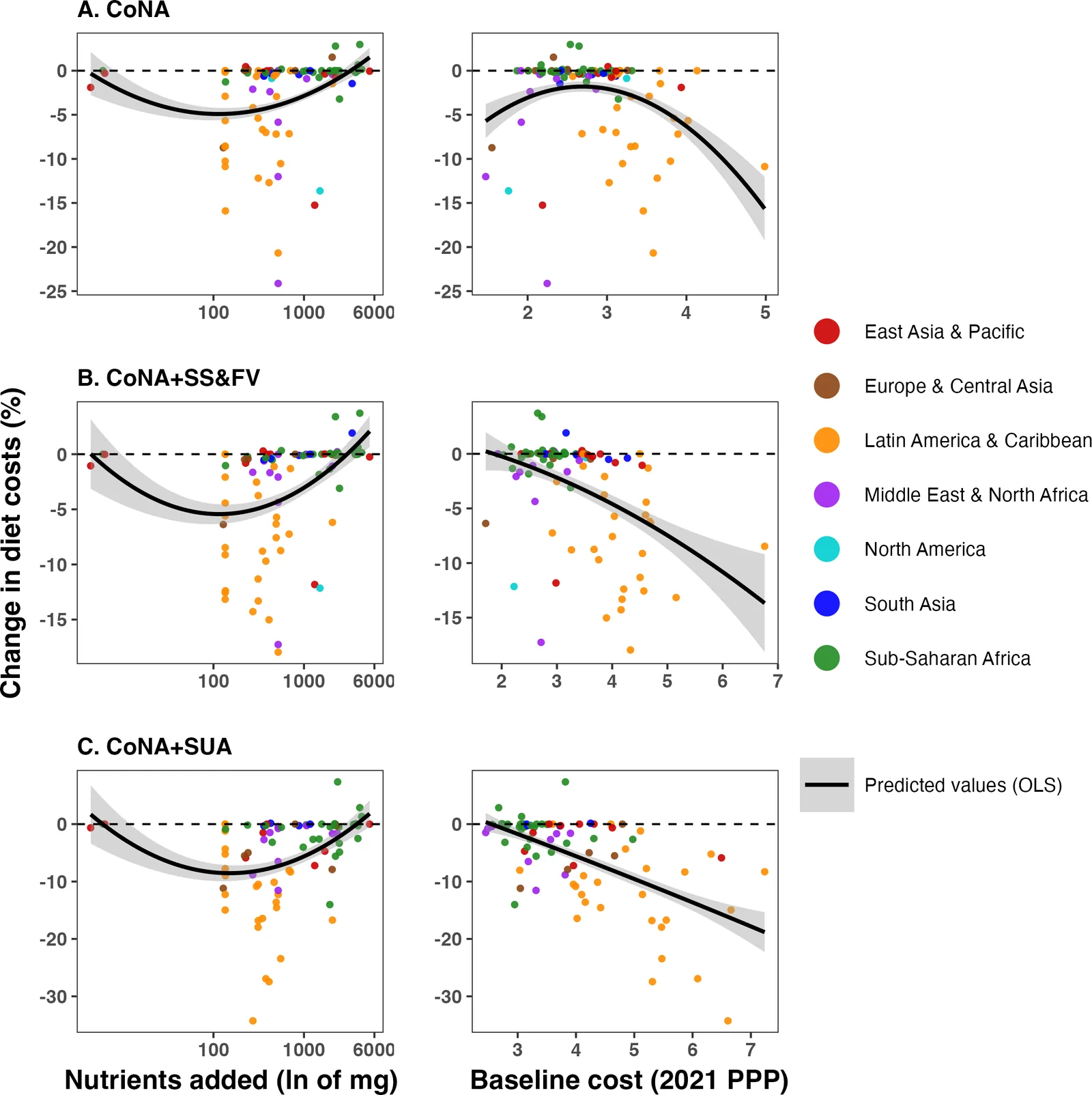
Diet cost reductions compared with combined fortification quantities and baseline modelled diet costs by country and region. Scatter plot values shown on the y-axis are median changes in country-level diet costs across all population subgroups, including each modelled diet scenario separately. Regions are from the World Bank Development Indicators. Black lines indicate predicted values from quadratic ordinary least squares (OLS) regressions with fixed effects for region and diet cost model, and grey shaded areas indicate 95% CIs. In the left-hand column, data shown on the x-axis are country-level totals in fortification amounts across all nutrients and food vehicles, expressed in mg of nutrient added per kg of food vehicle and shown on a log scale. In the right-hand column, data shown on the x-axis are country-level diet costs assuming no LSFF implementation. Cases with no feasible solution are omitted. CoNA, cost of nutrient adequacy; FV, fruits and vegetables; LSFF, large-scale food fortification; SS, starchy staples; SUA, Supply Utilization Accounts.

Several countries consistently exhibited the largest average reductions across all scenarios (online supplemental appendix figure A2). For example, in the basic CoNA scenario, LSFF reduced median diet costs most significantly in Qatar (mean=24.1%), Paraguay (20.7%) and Trinidad and Tobago (15.9%). In the CoNA-SS&FV scenario, LSFF reduced median diet costs most significantly in Paraguay (18.0%), Qatar (17.3%) and Uruguay (15.0%). Finally, in the CoNA-SUA scenario, LSFF resulted in large median reductions in diet costs of greater than 25% in Colombia (34.3%), Uruguay (27.5%) and Argentina (26.9%), while a further 17 countries had reductions between 10% and 25%. The same countries tended to experience relatively substantial reductions under all three scenarios, indicating that fortification would be especially effective in increasing economic access to nutrient-adequate diets in these countries. In cases where fortification led to diet cost increases for any given model and population subgroup, this may reflect inefficiently high fortification levels as implied in figure 5, especially for younger children whose reference values for nutrients like zinc are relatively low (see online supplemental appendix figure A3 for examples).

## Discussion

Our results reveal that implementation of existing LSFF standards leads to modest reductions in the cost of nutrient-adequate diets, with substantial variation across countries and sex-age groups, and with within-country differences further driven by specific food vehicles and nutrients more than others. The largest cost reductions were observed in diets that provide food group amounts that match current consumption patterns. In slightly more than one-third of modelled diets, LSFF implementation had no impact on diet costs, while diet costs rise in a smaller number of modelled diets. Differences in the direction and magnitude of change reflect variation in national food prices and fortification policies, including the number of food vehicles fortified, range of nutrients mandated, fortification levels, and the dietary assumptions embedded in the least-cost modelling.

Single-vehicle and single-nutrient analyses reveal that, in select cases, fortification of a single food vehicle can effectively reduce nutrient-adequate diet costs, while fortification with a single nutrient often had little or no effect, with exceptions for calcium and iron. Among food vehicles, wheat flour fortification was the most effective in reducing diet costs. Among nutrients, iron and calcium contributed the most to cost reductions, indicating that these nutrients were the most costly to obtain even from least-cost sources within the optimised nutrient-adequate diet. The addition of other single nutrients to the food supply may have meaningful public health benefits, but these did not appear to reduce the cost of nutrient-adequate diets. This indicates that single nutrient fortification of starchy staples and oils or inefficiently high fortification levels for nutrients with ULs may cause the optimisation model to include more expensive foods that allow the modelled diet to remain within all nutrient constraints. Fortification of multiple food vehicles with multiple nutrients at intermediate fortification levels is likely to avoid this problem as the role of any single food or nutrient is reduced.

The modelled diet cost reductions represent potential improvements in economic access to nutrient-adequate diets across a range of food group specifications. Compared with previous studies based on the basic CoNA analysis,^17,19^ which estimates the absolute lower-bound cost of achieving nutrient adequacy, this analysis introduced expanded scenarios that incorporate food group-level constraints. In particular, the CoNA-SUA scenario integrated large-scale consumption patterns derived from national SUA, addressing critiques of the basic CoNA model’s unrealistic assumptions by aligning diet modelling more closely with actual food supply distributions and cultural preferences. This scenario produced cost estimates that were higher than each of the other two scenarios but also exhibited larger cost reduction percentages with LSFF implementation. The elevated cost likely reflected energy shares for comparatively expensive food groups for which consumption often overshoots food-based dietary guidelines, for example, animal-source foods.^26^ The CoNA-SUA scenario also produced cost estimates that approach the global average CoHD based on food-based dietary guidelines.^20^ These findings suggest that implementation of LSFF policies could improve access to nutrient-adequate diets even under locally preferred food group consumption patterns. Moreover, our analysis of the changes in item inclusion dynamics further shows that when food group constraints are included, cost reductions occur without compromising overall diet quality, as fortified items generally replace their unfortified counterparts rather than altering the balance of the diet. Finally, results show that cost reduction effects are consistently greater for nutritionally vulnerable groups, namely women of reproductive age, adolescent boys and older men.

This analysis had several limitations. First, the ICP food price data are nationally representative average prices, which ensures consistency and robustness but may obscure important local-level variation in food availability and affordability. Similarly, LSFF implementation may not reduce diet costs for all consumers within a given country, as some households, such as those that primarily purchase food at informal markets, may not have retail access to items subject to fortification. This analysis also did not account for all costs of LSFF implementation, such as costs to industry for equipment, labour, training, management and administration, or costs to government related to monitoring and enforcement efforts. These costs were estimated in Friesen *et al*^7^ but were not capable of item-level mapping for diet cost modelling and were thus excluded from this analysis.

Second, this analysis was strictly limited to modelling of diet costs and did not allow for comparisons with reported diets or food spending patterns. As such, a complete accounting of how modelled diets and observed behaviours either align or differ was not included. While the modelled diets are nutritionally adequate, and while the CoNA-SUA scenario was designed to approximate national dietary patterns, modelled diets may not fully align with national food-based dietary guidelines in each country. Future research could explore how incorporating both nutrient requirements and recommended consumption patterns into diet models may affect the estimated economic cost, particularly in scenarios that reflect healthy, diverse diets. Additional work could also investigate whether underlying food price patterns or particular aspects of LSFF policies explain the wide variation in country-level cost reductions reported here. Future work should also assess how cost reductions due to LSFF might improve the affordability of nutrient-adequate diets by comparing modelled diet costs with household purchasing power and the cost of basic needs.

These modelling findings show that implementation of LSFF generally produces only moderate reductions in the cost of nutrient-adequate diets and may in some cases have no cost effect or modestly increase diet costs. The extent of cost reductions varied widely across countries, fortification programme design and extent, and population subgroups. The greatest potential reduction was observed where fortification levels were intermediate rather than high, baseline diet costs were relatively high, and modelled diets reflected national average consumption patterns, as well as among vulnerable populations with higher nutrient needs. These results underscore that LSFF alone cannot make diets affordable, but that carefully designed, context-specific programmes can contribute meaningfully to improving access to adequate nutrient intake. Estimates of the extent of local nutrient deficiencies are also necessary for more sophisticated quantification of the benefits of LSFF, as improved health outcomes may outweigh the potential cost increases associated with high levels of fortification in places where nutrient deficiencies are extensive. Crucially, this analysis did not attempt to account for the full range of health and social benefits from LSFF, which are substantial,^7^ but focused exclusively on retail food costs as a measure of food security and access to nutritious diets. Accordingly, this work should not be taken to suggest that improved implementation of existing LSFF policies would not result in benefits to human health and well-being.

Experts should consider LSFF to be one element of a broader nutrition strategy, which requires the involvement of policy makers in government offices of health, agriculture, economic development and other areas related to the regulation of the food industry. Fortification must also accompany other policy interventions to either improve household purchasing power or reduce the cost of nutritious foods in cases where prices are unusually high. Fortification policies should also be designed with regard to optimal fortification amounts, both to achieve local health goals and to avoid unintended excess intake of any given nutrient. Although the diets modelled here do not perfectly match real-world consumption, this analysis suggests that well-designed LSFF programmes may improve the affordability of nutrient-adequate diets. It also highlights the complementary role of fortification alongside broader food system or market-based efforts to expand access to healthy diets that fully align with food-based dietary guidelines.

## Supplementary material

10.1136/bmjph-2025-004613 online supplemental file 1

## Data Availability

Data are available upon reasonable request.
