# Supplementary material for: Impacts of large-scale food fortification on the cost of nutrient-adequate diets: a modelling study in 89 countries
Source: BMJ Public Health. 2026 Sep 28;4(3):e004613. doi: 10.1136/bmjph-2025-004613 (PMC13629995; doi:10.1136/bmjph-2025-004613)
Supplement: Supplementary file 1 [file bmjph-4-3-s001.docx]

**Appendix**

**Impacts of large-scale food fortification on the cost of nutrient-adequate diets: a modelling study in 89 countries**

| **Table A1. Nutrients included in all least-cost diet calculations.** | | |
| --- | --- | --- |
| **Panel A. Energy and macronutrients** | | |
| Nutrient | | Type of constraint |
|  | Energy | Target quantity |
|  | Protein | Range |
|  | Lipids | Range |
|  | Carbohydrate | Range |
|  |  |  |
| **Panel B. Micronutrients** | | |
|  | Calcium | Range |
|  | Choline | Range |
|  | Copper | Lower bound (pregnancy and lactation)  Range (all other subgroups) |
|  | Folate | Lower bound |
|  | Iron | Range |
|  | Magnesium | Lower bound |
|  | Manganese | Range |
|  | Niacin | Lower bound |
|  | Phosphorus | Range |
|  | Retinol | Upper bound |
|  | Riboflavin | Lower bound |
|  | Selenium | Range |
|  | Sodium | Upper bound |
|  | Thiamin | Lower bound |
|  | Vitamin A | Lower bound |
|  | Vitamin B5 | Lower bound |
|  | Vitamin B6 | Range |
|  | Vitamin B12 | Lower bound |
|  | Vitamin C | Range |
|  | Vitamin E | Range |
|  | Zinc | Range |

| **Table A2. Sample matching of retail items to fortification vehicles** | | | | |
| --- | --- | --- | --- | --- |
| Country | Item | Bread (Y/N) | Fortifiable (Y/N) | Vehicle name |
| Kazakhstan | Rice (1) | No | No | N/A |
| Kazakhstan | Rice (2) | No | No | N/A |
| Kazakhstan | Rice (3) | No | No | N/A |
| Kazakhstan | Rice (4) | No | No | N/A |
| Kazakhstan | Wheat flour (1) | No | Yes | Wheat flour |
| Kazakhstan | Wheat flour (2) | No | Yes | Wheat flour |
| Kazakhstan | Wheat (1) | No | No | N/A |
| Kazakhstan | Bread (1) | Yes | Yes | Wheat flour |
| Kazakhstan | Bread (2) | Yes | Yes | Wheat flour |
| Kazakhstan | Bread (3) | Yes | Yes | Wheat flour |
| Kazakhstan | Bread (4) | Yes | Yes | Wheat flour |
| Zimbabwe | Rice (1) | No | No | N/A |
| Zimbabwe | Rice (2) | No | No | N/A |
| Zimbabwe | Rice (3) | No | No | N/A |
| Zimbabwe | Rice (4) | No | No | N/A |
| Zimbabwe | Wheat flour (1) | No | Yes | Wheat flour |
| Zimbabwe | Bread (1) | Yes | Yes | Wheat flour |
| Zimbabwe | Bread (2) | Yes | Yes | Wheat flour |
| Zimbabwe | Bread (3) | Yes | Yes | Wheat flour |
| Zimbabwe | Bread (4) | Yes | Yes | Wheat flour |
| Note: Items names are generic versions to protect proprietary ICP item names and codes. Items are classified as fortifiable if matched to a food vehicle that is designated as subject to mandatory or voluntary fortification policies in the GFDx database. | | | | |

| **Table A3. Median, minimum, and maximum modeled diet costs with 0% and 90% LSFF implementation by model and region, including all 89 countries in analytical dataset.** | | | | | | | |
| --- | --- | --- | --- | --- | --- | --- | --- |
|  |  | CoNA | | CoNA+SS&FV | | CoNA+SUA | |
| Region | Cost  (2021 PPP) | 0% LSFF | 90% LSFF | 0% LSFF | 90% LSFF | 0% LSFF | 90% LSFF |
| East Asia & Pacific | Median | 2.74 | 2.70 | 3.38 | 3.34 | 4.46 | 4.37 |
|  | Minimum | 1.09 | 0.96 | 1.34 | 1.34 | 1.76 | 1.78 |
|  | Maximum | 4.82 | 4.67 | 5.47 | 5.33 | 11.73 | 11.73 |
| Europe & Central Asia | Median | 2.29 | 2.27 | 2.65 | 2.63 | 4.00 | 3.79 |
|  | Minimum | 0.90 | 0.72 | 1.00 | 0.80 | 1.54 | 1.40 |
|  | Maximum | 3.88 | 3.89 | 4.35 | 4.35 | 8.77 | 8.77 |
| Latin America & Caribbean | Median | 3.33 | 3.13 | 4.08 | 3.76 | 4.82 | 4.17 |
|  | Minimum | 1.69 | 1.40 | 1.87 | 1.69 | 1.84 | 1.66 |
|  | Maximum | 6.11 | 5.65 | 8.17 | 7.72 | 9.54 | 8.37 |
| Middle East & North Africa | Median | 2.20 | 2.10 | 2.62 | 2.54 | 3.07 | 2.95 |
|  | Minimum | 1.06 | 0.84 | 1.11 | 1.11 | 1.37 | 1.36 |
|  | Maximum | 3.47 | 3.41 | 4.07 | 4.07 | 4.61 | 4.41 |
| North America | Median | 2.47 | 2.34 | 2.83 | 2.70 | 10.89 | 8.91 |
|  | Minimum | 0.95 | 0.74 | 1.25 | 1.03 | 3.89 | 3.21 |
|  | Maximum | 4.03 | 3.97 | 4.63 | 4.37 | 16.94 | 16.94 |
| South Asia | Median | 2.70 | 2.68 | 3.41 | 3.41 | 3.48 | 3.47 |
|  | Minimum | 1.58 | 1.57 | 2.07 | 2.07 | 1.99 | 1.99 |
|  | Maximum | 3.89 | 3.89 | 5.05 | 5.05 | 5.06 | 5.09 |
| Sub-Saharan Africa | Median | 2.51 | 2.51 | 2.70 | 2.69 | 3.13 | 3.08 |
|  | Minimum | 1.28 | 1.28 | 1.31 | 1.31 | 1.51 | 1.51 |
|  | Maximum | 4.51 | 4.51 | 4.58 | 4.58 | 5.62 | 5.47 |
| World | Median | 2.70 | 2.65 | 3.09 | 3.01 | 3.60 | 3.44 |
|  | Minimum | 0.90 | 0.72 | 1.00 | 0.80 | 1.37 | 1.36 |
|  | Maximum | 6.11 | 5.65 | 8.17 | 7.72 | 16.94 | 16.94 |
| Note: Data shown are diet cost estimates calculated across 3 nutrient adequacy scenarios using retail food prices from 2021. World regions are from the World Bank Development Indicators. Diet costs are shown in 2021 international dollars (PPP). LSFF refers to large-scale food fortification. CoNA refers to diets meeting nutrient requirements; CoNA-SS&FV refers to diets that also meet basic healthy food group constraints for starchy staples, fruits, and vegetables; and CoNA-SUA refers to diets that align with national average consumption patterns at the food group level. | | | | | | | |

| **Table A4. Region and income group classifications for countries included in fortification analysis** | | | |
| --- | --- | --- | --- |
| **Country** | **ISO3** | **Region** | **Income group** |
| Antigua and Barbuda | ATG | Latin America & Caribbean | High income |
| Argentina | ARG | Latin America & Caribbean | Upper-middle income |
| Australia | AUS | East Asia & Pacific | High income |
| Bahrain | BHR | Middle East & North Africa | High income |
| Bangladesh | BGD | South Asia | Lower-middle income |
| Belize | BLZ | Latin America & Caribbean | Upper-middle income |
| Benin | BEN | Sub-Saharan Africa | Lower-middle income |
| Bolivia | BOL | Latin America & Caribbean | Lower-middle income |
| Brazil | BRA | Latin America & Caribbean | Upper-middle income |
| Burkina Faso | BFA | Sub-Saharan Africa | Lower income |
| Burundi | BDI | Sub-Saharan Africa | Lower income |
| Cabo Verde | CPV | Sub-Saharan Africa | Lower-middle income |
| Cameroon | CMR | Sub-Saharan Africa | Lower-middle income |
| Canada | CAN | North America | High income |
| Chad | TCD | Sub-Saharan Africa | Lower income |
| Chile | CHL | Latin America & Caribbean | High income |
| China | CHN | East Asia & Pacific | Upper-middle income |
| Colombia | COL | Latin America & Caribbean | Upper-middle income |
| Congo | COG | Sub-Saharan Africa | Lower-middle income |
| Costa Rica | CRI | Latin America & Caribbean | Upper-middle income |
| Cote d'Ivoire | CIV | Sub-Saharan Africa | Lower-middle income |
| Djibouti | DJI | Middle East & North Africa | Lower-middle income |
| Dominica | DMA | Latin America & Caribbean | Upper-middle income |
| Dominican Republic | DOM | Latin America & Caribbean | Upper-middle income |
| Ecuador | ECU | Latin America & Caribbean | Upper-middle income |
| El Salvador | SLV | Latin America & Caribbean | Lower-middle income |
| Eswatini | SWZ | Sub-Saharan Africa | Lower-middle income |
| Ethiopia | ETH | Sub-Saharan Africa | Lower income |
| Fiji | FJI | East Asia & Pacific | Upper-middle income |
| Gambia | GMB | Sub-Saharan Africa | Lower income |
| Ghana | GHA | Sub-Saharan Africa | Lower-middle income |
| Grenada | GRD | Latin America & Caribbean | Upper-middle income |
| Guinea | GIN | Sub-Saharan Africa | Lower income |
| Guyana | GUY | Latin America & Caribbean | Upper-middle income |
| Honduras | HND | Latin America & Caribbean | Lower-middle income |
| India | IND | South Asia | Lower-middle income |
| Indonesia | IDN | East Asia & Pacific | Lower-middle income |
| Iraq | IRQ | Middle East & North Africa | Upper-middle income |
| Jamaica | JAM | Latin America & Caribbean | Upper-middle income |
| Jordan | JOR | Middle East & North Africa | Upper-middle income |
| Kazakhstan | KAZ | Europe & Central Asia | Upper-middle income |
| Kenya | KEN | Sub-Saharan Africa | Lower-middle income |
| Kuwait | KWT | Middle East & North Africa | High income |
| Liberia | LBR | Sub-Saharan Africa | Lower income |
| Malawi | MWI | Sub-Saharan Africa | Lower income |
| Malaysia | MYS | East Asia & Pacific | Upper-middle income |
| Mali | MLI | Sub-Saharan Africa | Lower income |
| Mexico | MEX | Latin America & Caribbean | Upper-middle income |
| Moldova | MDA | Europe & Central Asia | Upper-middle income |
| Mongolia | MNG | East Asia & Pacific | Lower-middle income |
| Morocco | MAR | Middle East & North Africa | Lower-middle income |
| Mozambique | MOZ | Sub-Saharan Africa | Lower income |
| Nepal | NPL | South Asia | Lower-middle income |
| Netherlands | NLD | Europe & Central Asia | High income |
| New Zealand | NZL | East Asia & Pacific | High income |
| Nicaragua | NIC | Latin America & Caribbean | Lower-middle income |
| Niger | NER | Sub-Saharan Africa | Lower income |
| Nigeria | NGA | Sub-Saharan Africa | Lower-middle income |
| Oman | OMN | Middle East & North Africa | High income |
| Pakistan | PAK | South Asia | Lower-middle income |
| Panama | PAN | Latin America & Caribbean | High income |
| Paraguay | PRY | Latin America & Caribbean | Upper-middle income |
| Peru | PER | Latin America & Caribbean | Upper-middle income |
| Philippines | PHL | East Asia & Pacific | Lower-middle income |
| Qatar | QAT | Middle East & North Africa | High income |
| Rwanda | RWA | Sub-Saharan Africa | Lower income |
| Saint Kitts and Nevis | KNA | Latin America & Caribbean | High income |
| Saint Lucia | LCA | Latin America & Caribbean | Upper-middle income |
| Saint Vincent and the Grenadines | VCT | Latin America & Caribbean | Upper-middle income |
| Saudi Arabia | SAU | Middle East & North Africa | High income |
| Senegal | SEN | Sub-Saharan Africa | Lower-middle income |
| Sierra Leone | SLE | Sub-Saharan Africa | Lower income |
| South Africa | ZAF | Sub-Saharan Africa | Upper-middle income |
| Sri Lanka | LKA | South Asia | Lower-middle income |
| Sudan | SDN | Sub-Saharan Africa | Lower income |
| Suriname | SUR | Latin America & Caribbean | Upper-middle income |
| Tanzania | TZA | Sub-Saharan Africa | Lower-middle income |
| Thailand | THA | East Asia & Pacific | Upper-middle income |
| Togo | TGO | Sub-Saharan Africa | Lower income |
| Trinidad and Tobago | TTO | Latin America & Caribbean | High income |
| Uganda | UGA | Sub-Saharan Africa | Lower income |
| United Arab Emirates | ARE | Middle East & North Africa | High income |
| United Kingdom | GBR | Europe & Central Asia | High income |
| United States of America | USA | North America | High income |
| Uruguay | URY | Latin America & Caribbean | High income |
| Uzbekistan | UZB | Europe & Central Asia | Lower-middle income |
| Viet Nam | VNM | East Asia & Pacific | Lower-middle income |
| Zambia | ZMB | Sub-Saharan Africa | Lower income |
| Zimbabwe | ZWE | Sub-Saharan Africa | Lower-middle income |

| **Table A5. Data availability and fortification coverage (n = country).** | | | | | |
| --- | --- | --- | --- | --- | --- |
|  | | | | | |
| **Panel A. Data coverage** | | | | | |
| International Comparisons Program (ICP) 2021^20^ | | | | 173 | |
| Global Fortification Data Exchange (GFDx)^21^ | | | | 154 | |
| FAO Supply Utilization Accounts (SUA)^19^ | | | | 190 | |
| Final analytical sample | | | | 89 | |
|  | | | | | |
| **Panel B. Fortification policies by nutrient and food vehicle** | | | | | |
| **Nutrient** | **Food Vehicle** | | | |  |
|  | Wheat flour | Maize flour | Oil | Rice | Any vehicle |
| Iron | 82 | 18 | 0 | 10 | 82 |
| Zinc | 30 | 11 | 0 | 6 | 34 |
| Calcium | 23 | 2 | 0 | 2 | 23 |
| Vitamin B12 | 21 | 10 | 0 | 6 | 25 |
| Folate | 71 | 17 | 0 | 9 | 71 |
| Niacin | 59 | 16 | 0 | 10 | 61 |
| Riboflavin | 59 | 15 | 0 | 4 | 61 |
| Thiamin | 61 | 16 | 0 | 11 | 63 |
| Vitamin B6 | 16 | 9 | 0 | 5 | 20 |
| Vitamin A | 21 | 10 | 36 | 3 | 43 |
| Vitamin E | 0 | 0 | 2 | 1 | 3 |
| Selenium | 1 | 0 | 0 | 1 | 2 |
| Any nutrient | 86 | 18 | 37 | 12 | 89 |
| Note: Fortification policies represent unique country, nutrient, and food vehicle combinations, including both mandatory and voluntary standards as documented in GFDx. | | | | | |

| **Table A6. Diet costs with alternative rates of LSFF implementation and premix cost pass-through** | | | | |
| --- | --- | --- | --- | --- |
| A. CoNA | | | | |
|  | Base | 90%LSFF + 50% PT | 50% LSFF + 100% PT | 90% LSFF + 100% PT |
| East Asia & Pacific | 2.74 (0.93) | 2.72 (1.02) | 2.73 (1.02) | 2.72 (1.02) |
| Europe & Central Asia | 2.24 (0.70) | 2.24 (0.79) | 2.23 (0.75) | 2.24 (0.79) |
| Latin America & Caribbean | 3.32 (0.87) | 3.10 (0.89) | 3.17 (0.87) | 3.10 (0.89) |
| Middle East & North Africa | 2.20 (0.71) | 2.09 (0.81) | 2.14 (0.74) | 2.09 (0.80) |
| North America | 2.30 (1.47) | 2.12 (1.65) | 2.13 (1.65) | 2.13 (1.65) |
| South Asia | 2.72 (0.65) | 2.70 (0.68) | 2.68 (0.68) | 2.70 (0.68) |
| Sub-Saharan Africa | 2.48 (0.72) | 2.48 (0.72) | 2.48 (0.72) | 2.48 (0.72) |
|  |  |  |  |  |
| B. CoNA-SS&FV | | | | |
|  | Base | 90%LSFF + 50% PT | 50% LSFF + 100% PT | 90% LSFF + 100% PT |
| East Asia & Pacific | 3.41 (1.04) | 3.36 (1.13) | 3.38 (1.11) | 3.36 (1.13) |
| Europe & Central Asia | 2.63 (1.10) | 2.63 (1.13) | 2.62 (1.13) | 2.63 (1.13) |
| Latin America & Caribbean | 4.08 (1.12) | 3.74 (1.10) | 3.82 (1.07) | 3.74 (1.10) |
| Middle East & North Africa | 2.63 (0.80) | 2.52 (0.81) | 2.56 (0.76) | 2.53 (0.81) |
| North America | 2.70 (1.32) | 2.49 (1.52) | 2.49 (1.50) | 2.49 (1.52) |
| South Asia | 3.36 (1.08) | 3.39 (1.06) | 3.35 (1.06) | 3.39 (1.06) |
| Sub-Saharan Africa | 2.69 (0.80) | 2.69 (0.81) | 2.69 (0.80) | 2.69 (0.81) |
|  |  |  |  |  |
| C. CoNA-SUA | | | | |
|  | Base | 90%LSFF + 50% PT | 50% LSFF + 100% PT | 90% LSFF + 100% PT |
| East Asia & Pacific | 4.03 (1.41) | 3.92 (1.46) | 3.92 (1.47) | 3.92 (1.46) |
| Europe & Central Asia | 4.06 (1.49) | 3.84 (1.67) | 3.93 (1.57) | 3.84 (1.67) |
| Latin America & Caribbean | 4.73 (1.69) | 4.09 (1.35) | 4.34 (1.53) | 4.09 (1.35) |
| Middle East & North Africa | 3.04 (1.12) | 2.93 (0.96) | 2.99 (1.02) | 2.93 (0.96) |
| North America | 10.84 (6.51) | 6.92 (7.51) | 8.14 (6.87) | 6.92 (7.51) |
| South Asia | 3.45 (0.87) | 3.45 (0.88) | 3.45 (0.87) | 3.45 (0.88) |
| Sub-Saharan Africa | 3.09 (0.80) | 3.06 (0.83) | 3.06 (0.83) | 3.06 (0.83) |
|  |  |  |  |  |
| Note: Data shown are median diet cost estimates (interquartile ranges) in 2021 international dollars (PPP). Base = No fortification; Fort 1 = sensitivity analysis with 90% LSFF implementation and 50% pass-through of premix costs; Fort 2 = sensitivity analysis with 50% LSFF implementation and 100% pass-through of premix costs; and Fort 3 = main fortification analysis with 90% LSFF implementation and 100% pass-through of premix costs. CoNA refers to diets meeting nutrient requirements; CoNA-SS&FV refers to diets that also meet basic healthy food group constraints for starchy staples, fruits, and vegetables; and CoNA-SUA refers to diets that align with national average consumption patterns at the food group level. | | | | |

| **Table A7. Modeled diet cost scenarios with no feasible solution by country and subgroup** | | |
| --- | --- | --- |
| **Country** | **Subgroup** | **Scenario** |
| Australia | Male (11 to 14) | CoNA-SUA (no LSFF) |
| Australia | Male (11 to 14) | CoNA-SUA (with LSFF) |
| Bangladesh | Male (71 to 79) | CoNA-SUA (no LSFF) |
| Bangladesh | Male (71 to 79) | CoNA-SUA (with LSFF) |
| Canada | Female (11 to 14) | CoNA-SUA (no LSFF) |
| Canada | Female (11 to 14) | CoNA-SUA (with LSFF) |
| Canada | Female (15 to 17) | CoNA-SUA (no LSFF) |
| Canada | Female (15 to 17) | CoNA-SUA (with LSFF) |
| Canada | Female (18 to 24) | CoNA-SUA (no LSFF) |
| Canada | Female (18 to 24) | CoNA-SUA (with LSFF) |
| Canada | Female (25 to 50) | CoNA-SUA (no LSFF) |
| Canada | Female (25 to 50) | CoNA-SUA (with LSFF) |
| Canada | Female (51 to 70) | CoNA-SUA (no LSFF) |
| Canada | Female (51 to 70) | CoNA-SUA (with LSFF) |
| Canada | Female (7 to 10) | CoNA-SUA (no LSFF) |
| Canada | Female (7 to 10) | CoNA-SUA (with LSFF) |
| Canada | Female (71 to 79) | CoNA-SUA (no LSFF) |
| Canada | Female (71 to 79) | CoNA-SUA (with LSFF) |
| Canada | Male (11 to 14) | CoNA-SUA (no LSFF) |
| Canada | Male (11 to 14) | CoNA-SUA (with LSFF) |
| Canada | Male (15 to 17) | CoNA-SUA (no LSFF) |
| Canada | Male (15 to 17) | CoNA-SUA (with LSFF) |
| El Salvador | Female (11 to 14) | CoNA-SUA (no LSFF) |
| El Salvador | Female (11 to 14) | CoNA-SUA (with LSFF) |
| El Salvador | Female (15 to 17) | CoNA-SUA (no LSFF) |
| El Salvador | Female (15 to 17) | CoNA-SUA (with LSFF) |
| El Salvador | Female (18 to 24) | CoNA-SUA (no LSFF) |
| El Salvador | Female (18 to 24) | CoNA-SUA (with LSFF) |
| El Salvador | Female (25 to 50) | CoNA-SUA (no LSFF) |
| El Salvador | Female (25 to 50) | CoNA-SUA (with LSFF) |
| El Salvador | Female (4 to 6) | CoNA-SUA (no LSFF) |
| El Salvador | Female (4 to 6) | CoNA-SUA (with LSFF) |
| El Salvador | Female (51 to 70) | CoNA-SUA (no LSFF) |
| El Salvador | Female (51 to 70) | CoNA-SUA (with LSFF) |
| El Salvador | Female (7 to 10) | CoNA-SUA (no LSFF) |
| El Salvador | Female (7 to 10) | CoNA-SUA (with LSFF) |
| El Salvador | Female (71 to 79) | CoNA-SUA (no LSFF) |
| El Salvador | Female (71 to 79) | CoNA-SUA (with LSFF) |
| El Salvador | Lactating (19 to 30) | CoNA-SUA (no LSFF) |
| El Salvador | Lactating (19 to 30) | CoNA-SUA (with LSFF) |
| El Salvador | Lactating (31 to 50) | CoNA-SUA (no LSFF) |
| El Salvador | Lactating (31 to 50) | CoNA-SUA (with LSFF) |
| El Salvador | Lactating (18 and under) | CoNA-SUA (no LSFF) |
| El Salvador | Lactating (18 and under) | CoNA-SUA (with LSFF) |
| El Salvador | Male (11 to 14) | CoNA-SUA (no LSFF) |
| El Salvador | Male (11 to 14) | CoNA-SUA (with LSFF) |
| El Salvador | Male (15 to 17) | CoNA-SUA (no LSFF) |
| El Salvador | Male (15 to 17) | CoNA-SUA (with LSFF) |
| El Salvador | Male (18 to 24) | CoNA-SUA (no LSFF) |
| El Salvador | Male (18 to 24) | CoNA-SUA (with LSFF) |
| El Salvador | Male (25 to 50) | CoNA-SUA (no LSFF) |
| El Salvador | Male (25 to 50) | CoNA-SUA (with LSFF) |
| El Salvador | Male (4 to 6) | CoNA-SUA (no LSFF) |
| El Salvador | Male (4 to 6) | CoNA-SUA (with LSFF) |
| El Salvador | Male (51 to 70) | CoNA-SUA (no LSFF) |
| El Salvador | Male (51 to 70) | CoNA-SUA (with LSFF) |
| El Salvador | Male (7 to 10) | CoNA-SUA (no LSFF) |
| El Salvador | Male (7 to 10) | CoNA-SUA (with LSFF) |
| El Salvador | Male (71 to 79) | CoNA-SUA (no LSFF) |
| El Salvador | Male (71 to 79) | CoNA-SUA (with LSFF) |
| El Salvador | Pregnant (19 to 30) | CoNA-SUA (no LSFF) |
| El Salvador | Pregnant (19 to 30) | CoNA-SUA (with LSFF) |
| El Salvador | Pregnant (31 to 50) | CoNA-SUA (no LSFF) |
| El Salvador | Pregnant (31 to 50) | CoNA-SUA (with LSFF) |
| El Salvador | Pregnant (18 and under) | CoNA-SUA (no LSFF) |
| El Salvador | Pregnant (18 and under) | CoNA-SUA (with LSFF) |
| United States of America | Female (11 to 14) | CoNA-SUA (no LSFF) |
| United States of America | Female (25 to 50) | CoNA-SUA (no LSFF) |
| United States of America | Female (7 to 10) | CoNA-SUA (no LSFF) |
| United States of America | Male (11 to 14) | CoNA-SUA (no LSFF) |
| Note: CoNA-SUA refers to diets that align with national average consumption patterns at the food group level. LSFF refers to large-scale food fortification. | | |

**Figure A1. Food group inclusion without and with fortification relative to Healthy Diet Basket reference intakes**

**Note:** Data shown are average kilocalories per day included in modeled diets by food group, expressed as the ratio of reference intakes from the Healthy Diet Basket^13^. Reference intakes are adjusted proportionally for each sex-age subgroup’s total energy needs. Dashed horizontal lines indicate where food group consumption is equal to reference intakes. Ratios are calculated at the model-subgroup level and then converted to the overall averages shown here. CoNA refers to diets meeting nutrient requirements; CoNA-SS&FV refers to diets that also meet basic healthy food group constraints for starchy staples, fruits, and vegetables; and CoNA-SUA refers to diets that align with national average consumption patterns at the food group level. The suffix “-fort” indicates that 90% LSFF implementation has been included in the diet cost modeling scenario.

**Figure A2. Variation in country-level diet cost reductions with improved scenario by scenario and number of fortification policies.**

**Note:** Data shown are changes in diet costs from countries with non-zero change in diet costs under LSFF implementation. Boxplots indicate the distribution of diet cost changes within each country across 22 sex-age subgroups. Negative values indicate reduced costs with LSFF while positive values indicate increased costs with LSFF. Shading within each box indicates the number of unique fortification policies documented in each country, e.g. the total number of policies per food vehicle and nutrient combination. Y-axis labels are ISO3 country codes. CoNA refers to diets meeting nutrient requirements; CoNA-SS&FV refers to diets that also meet basic healthy food group constraints for starchy staples, fruits, and vegetables; and CoNA-SUA refers to diets that align with national average consumption patterns at the food group level. Outliers are omitted from all boxplots using the Tukey standard (1.5 x IQR).

**Figure A3. Examples of food item inclusion with and without fortification under CoNA+SUA scenario for girls aged 4y to 6y.**

**Note:** Data shown are cost per day in 2021 international dollars (PPP) to purchase items included in diets meeting CoNA+SUA with and without fortification for girls aged 4 to 6. Costs are shown for three selected countries where fortification increases average diet costs. CoNA-SUA refers to diets that align with national average consumption patterns at the food group level.

**Figure A4. Fortification premix costs as a share of retail prices by food vehicle**

**Note:** Data shown are premix costs as estimated in Friesen et al. (2026) as a percentage of retail food prices for foods matched to fortification vehicles at the country level. Prices and premix costs are each specified in 2021 international dollars (PPP) per kilogram of fortified food vehicle. Outliers are omitted from all boxplots using the Tukey standard (1.5 x IQR).

**Figure A5. Percentage change in diet costs by country income group (all countries and population subgroups)**

**Note:** Data shown are changes in diet costs under LSFF implementation, with all 89 sample countries included in the sample. Boxplots indicate the distribution of diet cost changes within each country across 22 sex-age subgroups. Negative values indicate reduced costs with LSFF while positive values indicate increased costs with LSFF. CoNA refers to diets meeting nutrient requirements; CoNA-SS&FV refers to diets that also meet basic healthy food group constraints for starchy staples, fruits, and vegetables; and CoNA-SUA refers to diets that align with national average consumption patterns at the food group level. Outliers are omitted from all boxplots using the Tukey standard (1.5 x IQR).
